# Successful BCMA CAR-T Therapy for Multiple Myeloma With Central Nervous System Involvement Manifesting as Cauda Equina Syndrome—A Wandering Road to Remission

**DOI:** 10.3389/fonc.2021.755584

**Published:** 2021-11-17

**Authors:** Yiyun Wang, Linqin Wang, Yifan Zeng, Ruimin Hong, Cheng Zu, Elaine Tan Su Yin, Houli Zhao, Guoqing Wei, Li Yang, Aiyun Jin, Yongxian Hu, He Huang

**Affiliations:** ^1^ Bone Marrow Transplantation Center, the First Affiliated Hospital, Zhejiang University School of Medicine, Hangzhou, China; ^2^ Institute of Hematology, Zhejiang University, Hangzhou, China; ^3^ Zhejiang Province Engineering Laboratory for Stem Cell and Immunity Therapy, Hangzhou, China; ^4^ Zhejiang Laboratory for Systems & Precision Medicine, Zhejiang University Medical Center, Hangzhou, China; ^5^ Department of Neurology, The First Affiliated Hospital of Wenzhou Medical University, Wenzhou, China

**Keywords:** multiple myeloma, central nervous system, chimeric antigen receptor (CAR T), immunotherapy, cauda equina syndrome

## Abstract

Multiple myeloma (MM) with central nervous system (CNS) involvement is rare with only 1% incidence. So far, there is no standard or effective treatment for CNS MM, and the expected survival time is fewer than 6 months. Here, we report a case of MM with CNS involvement presented with cauda equina syndrome (CES) who achieved complete remission after anti-B-cell maturation antigen (BCMA) chimeric antigen receptor T (CAR-T) cell therapy (Chictr.org.cn, ChiCTR1800017404). The expansion of BCMA CAR-T cells was observed in both peripheral blood (PB) and cerebrospinal fluid (CSF). The CAR-T cells peaked at 2.4 × 10^6^/l in CSF at day 8 and 4.1 × 10^9^/l in PB at day 13. The peak concentration of interleukin (IL)-6 in CSF was detected 3 days earlier, and almost five times higher than that in PB. Next, morphological analysis confirmed the elimination of nucleated cells in CSF 1 month after CAR-T cell treatment from 300 cells/μl, and the patient achieved functional recovery with regressed lesion shown in PET-CT. The case demonstrated that BCMA CAR-T cells are effective and safe in this patient population.

## Introduction

Multiple myeloma (MM) is a clonal plasma cell malignancy which accounted for 10% of hematologic malignancies. In MM, extramedullary diseases in the central nervous system (CNS) are rare and are diagnosed in less than 1% of MM patients (1). The mechanism of CNS infiltration remains unclear—two hypotheses were suggested: the hematogenous spread of malignant plasma cells and the direct invasion from proximal lesions (2). So far, there is no standard or effective regimen for CNS MM. Conventional treatment methods like systemic chemotherapy, local radiotherapy, and intrathecal injection were used to treat MM patients (1, 3, 4). Nonetheless, the MM patients’ prognosis with CNS infiltration is abysmal, and the expected survival time is fewer than 6 months (1–6).

On the other hand, chimeric antigen receptor T (CAR-T) cell therapy has become a promising method to treat hematological malignancies (7–9). It is noteworthy that CNS involvement was considered one of the exclusion criteria for CAR-T clinical trials concerning severe local severe cytokine release syndrome (CRS) and immune effector cell-associated neurotoxicity syndrome (ICANS) in early time (10–12). However, an increasing number of reports suggested that CAR-T is effective and safe to ALL and lymphoma patients (13, 14). According to previous studies, anti-B-cell maturation antigen (BCMA) CAR-T in MM has achieved CR rates higher than 80%, but the efficacy of BCMA CAR-T in MM CNS patients has not been reported yet (15–19).

In this study, we report a case of refractory/relapsed MM with CNS involvement, manifesting as cauda equina syndrome (CES), and demonstrate the safety and effectiveness of BCMA CAR-T therapy in this patient population.

## Methods

### Patient

The patient was a 60-year-old man diagnosed with IgA/λ MM, positive for monoclonal IgH gene rearrangement, 1q21 amplification, and P53 mutation. He was given 3 cycles of chemotherapy with bortezomib, cyclophosphamide, and dexamethasone (VCD) and 3 cycles of bortezomib, lenalidomide, and dexamethasone (VRD). PET-CT scan showed a new osteolytic lesion in the left transverse process of the 7th thoracic vertebra which extended into the spinal canal. Accordingly, he received 7 cycles of local radiation combined with 15 cycles of chemotherapy (3 cycles of bortezomib + dexamethasone, etoposide, doxorubicin, cisplatin (DEAP); 10 cycles of melphalan, cyclophosphamide, and prednisone (MCP); and 2 cycles of dexamethasone, etoposide, cyclophosphamide, cisplatin (DECP). At that time, the patient achieved complete remission (CR) in bone marrow confirmed by morphological examination and flow cytometry, as well as negative serum and urine immunofixation, while the extramedullary lesion showed only partial regression shown by PET-CT. Four months later, he complained of pain and weakness in bilateral lower limbs accompanied by urinary incontinence and was diagnosed with secondary CES, which is a rarely reported complication of MM (20). He was therefore enrolled in BCMA CAR-T therapy trial (Chictr.org.cn, ChiCTR1800017404, details regarding the design of this trial are accessible at https://www.chictr.org.cn/showproj.aspx?proj=28864) after the approval by the ethics committee of the First Affiliated Hospital of Zhejiang University.

### BCMA CAR-T Cell Generation, Therapy, and Detection

**Figure 1:**
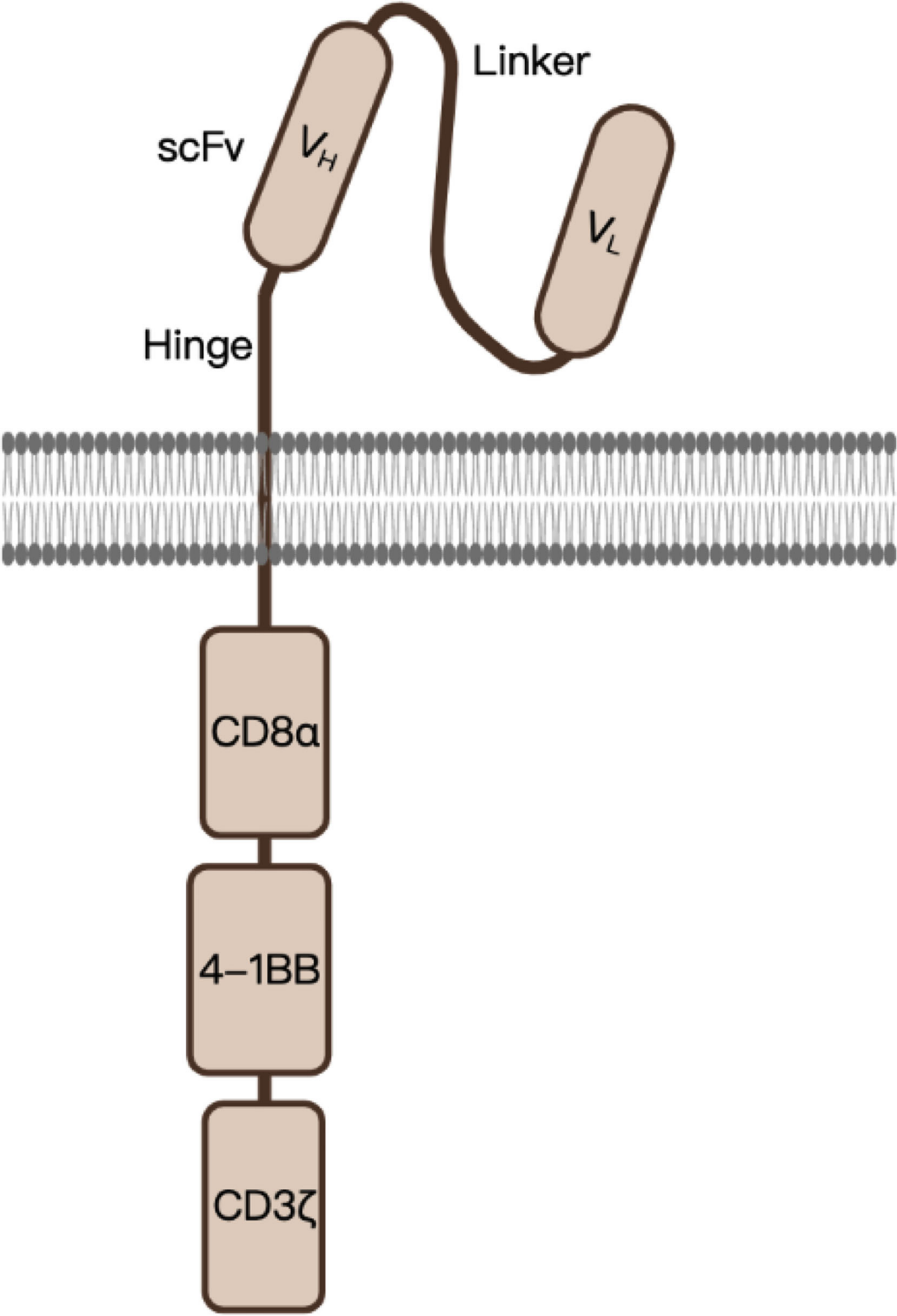
A schematic diagram of anti-BCMA CAR design. scFv, single-chain variable fragment, serving as the BCMA-recognition domain; CD8α, in which the hinge (transmembrane domain) was derived; 4-1BB, co-stimulatory domain which enhances the function of CAR; CD3ζ, serves as the signal-transduction domain.

The single-chain fragment variable (scFv) sequence of BCMA CAR was obtained from a murine hybridoma cell line raised against BCMA. In addition to the scFv, a 4-1BB co-stimulatory domain and a CDζ3-signaling domain were inserted into a lentiviral vector as well. The construct of this second-generation anti-BCMA CAR is shown in **Figure 1**.

The peripheral blood mononuclear cells were obtained from the patient by leukapheresis. The blood cells were transduced with BCMA CAR using lentivirus. He received a fludarabine- (30 mg/m^2^, day -4 to -2) and cyclophosphamide- (500 mg/m^2^, day -3 to -2) based lymphodepletion regimen before CAR-T cell infusion. After preconditioning chemotherapy, he received BCMA CAR-T cells at a dose of 7.1 × 10^6^/kg. Furthermore, an Ommaya reservoir was installed to obtain CSF samples once a day for consecutive 4 weeks. The grading of CRS was based on the Penn grading scale (8), and the grading of neurotoxicity was based on the Common Terminology Criteria for Adverse Events 5.0 (CTCAE 5.0) (21). The patient’s response was assessed 1 month after CAR-T therapy. Subsequently, the patient’s condition and MRD detection were being followed up in outpatient departments at 2, 3, 6, 12, 18, 24, 36, and 48 months. The expansions of *in vivo* CAR-T cells in the peripheral blood and CSF were continuously detected by flow cytometry. The methods to assess the treatment response included morphological analysis, flow cytometry, and MRI.

## Results and Discussion

**Figure 2:**
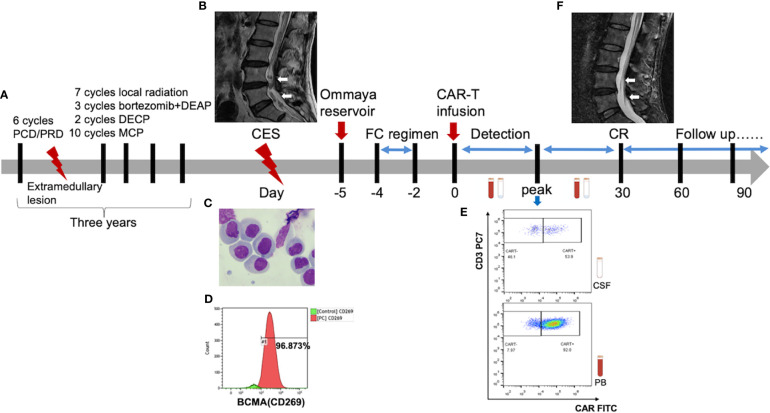
**(A)** The flowchart of patient’s treatment history and CAR-T therapy. **(B)** Pretreatment lumbar MRI showed multiple diffuse abnormal signal nods in the cauda equina at lumbar and caudal level. **(F)** The lesion disappeared in the post-treatment imaging, indicating a complete remission (CR) [high-resolution T2-weighted image]. **(C)** Abnormal plasma cells were observed under the microscope. There were 300 nucleated cells/μL in the cerebrospinal fluid of which 35% were plasmablast and 54% were proplasmacyte. **(D)** The flow cytometry indicated that 96.873% of the plasma cells were expressed with BCMA antigens. **(E)** The CAR-T cells peaked 53.9% in CSF at day 8 and 92.0% in PB at day 13.

The flowchart of the patient’s treatment history and CAR-T therapy is shown in **Figure 2A**. The infiltration of MM into CNS was confirmed by enhanced lumbar MRI (**Figure 2B**) and CSF examination. Morphological analysis further revealed the presence of 300 nucleated cells/μl (35% plasmablasts and 54% proplasmacytes) in CSF (**Figure 2C**) and 96.873% of plasma cells expressing BCMA (**Figure 2D**). Therefore, he was enrolled in the clinical trial of BCMA CAR-T cell therapy. To assess the response continually and safely, the patient was installed with an Ommaya reservoir. After a preconditioning chemotherapy, he received CAR-T cells at a dose of 7.1 × 10^6^/kg.

**Figure 3:**
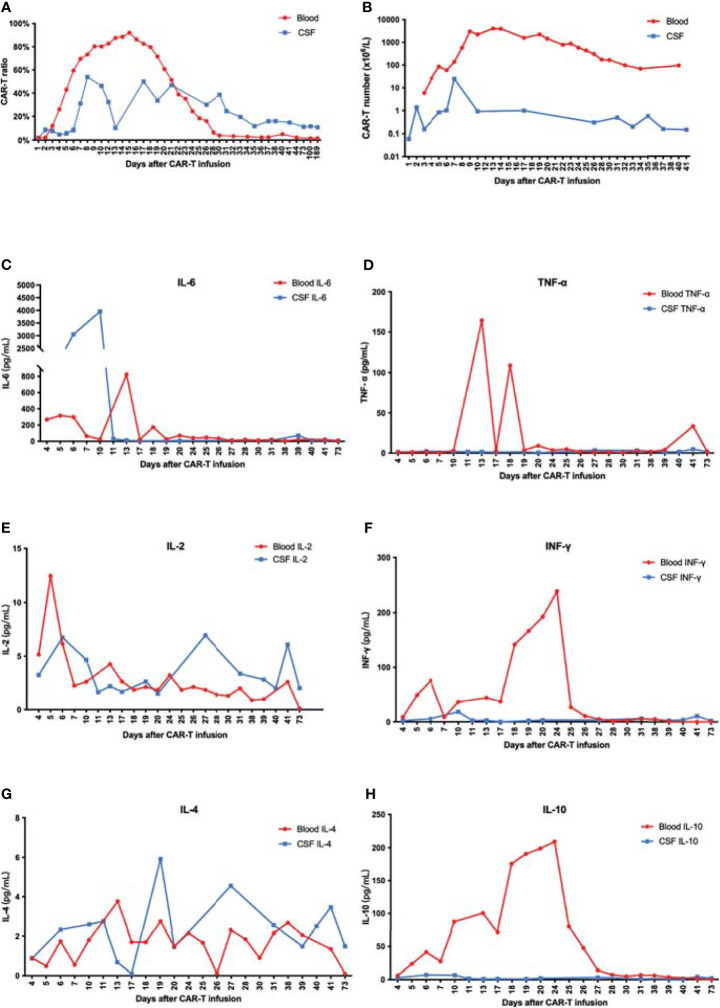
**(A)** The proportion of CAR-T cells in CD3-positive cells increased and reached a peak at 7 or 15 days after CAR-T infusion. **(B)** The number of CAR-T cells in peripheral blood far exceeded that in CSF. **(C–H)** IL-6 increased rapidly in CSF and was significantly higher than that in blood. There was no significant difference of IL-2 and IL-4 between peripheral blood and CSF. Moreover, IL-10, TNF-α, and IFN-γ in peripheral blood significantly exceeded that in CSF.

**Figure 4:**
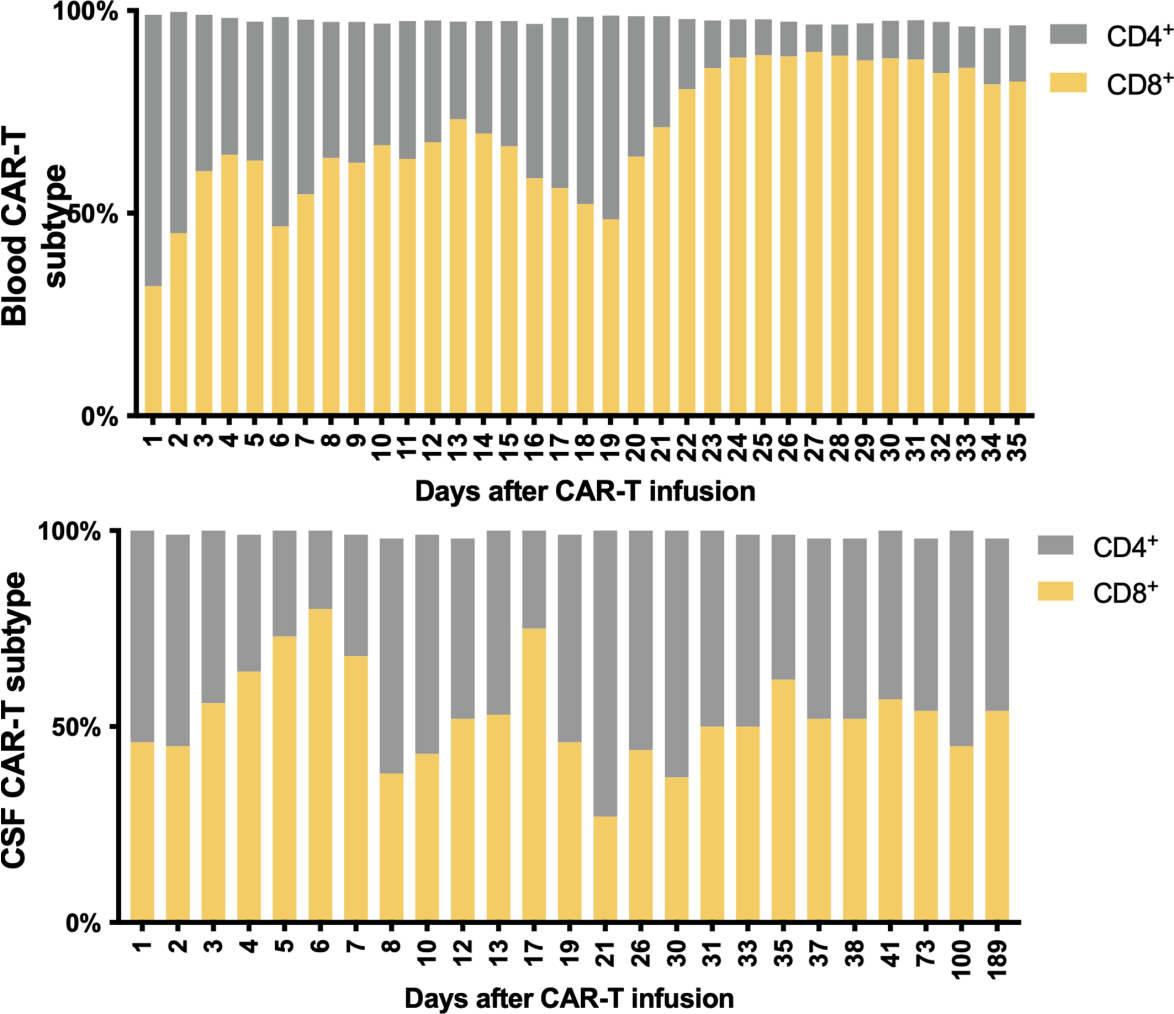
Subtypes of CAR-T cells in peripheral blood and CSF. CD8^+^ cells were predominant in the PB after CAR-T therapy, while it returned to normal CSF proportions in 7 days.

As a result, the patient had a high fever (38°C) 8 h after CAR-T cell infusion, indicating CRS onset. In the following weeks, the proliferation of CAR-T cells was observed in both peripheral blood (PB) and CSF. The CAR-T cells peaked at 2.4 × 10^6^/l in CSF at day 8 and 4.1 × 10^9^/l in PB at day 13 (**Figures 2E**, **3A, B**). Moreover, CD8^+^ cells were predominant in the PB after infusion, while it returned to normal proportion in CSF in 7 days (**Figure 4**).

The predominance of CD8^+^ CAR-T after infusion is in accord with the findings of previous publications (16, 22). These results indicate that CD8^+^ CAR-T cells may play a more central role in the elimination of tumor cells. The predominance was not as significant in CSF as in PB, which suggests that the penetration of CD4^+^ and CD8^+^ CAR-T cells across the BBB may be regulated by discrete mechanisms. Several trials found that an appropriately defined CD4^+^/CD8^+^ ratio can improve the efficacy of CAR-T cells (23, 24). Thus, the penetration ability should be taken into consideration when a preferred ratio was defined for the treatment of CNS-infiltrated malignancies.

Along with cell proliferation, cytokines increased rapidly. Remarkably, IL-6 in CSF increased fast to 3,953.44 pg/ml at day 10 and reached a peak of 823.11 pg/ml in PB at day 13. The peak of IL-6 in CSF was observed 3 days earlier and almost five times higher than that in PB. Meanwhile, the IL-10, TNF-α, and IFN-γ levels were much higher in PB, suggesting a major difference in the mechanisms of cytokine secretion between these two environments (**Figures 3C–H**).

To our surprise, the patient merely suffered a mild grade 2 systemic CRS without any manifestation of neurotoxicity. Zhang et al. reported a similar patient who suffered relatively severe neurotoxicity, including headache, lethargy, chemosis, stiff neck, aphasia, pupil asymmetry with loss of light reflex, and obtundation which further developed into stupor (25). Such a huge gap in the severity of neurotoxicity resulted from, we suppose, the different locations of CNS lesions. Our patient’s primary lesion was sited at the cauda equina, while the patient in the report by Zhang et al. had lesions located in the occipital lobe, thoracolumbar spine, and leptomeninges of the brainstem, which may account for the symptoms of impaired consciousness and cranial nerves.

**Figure 5:**
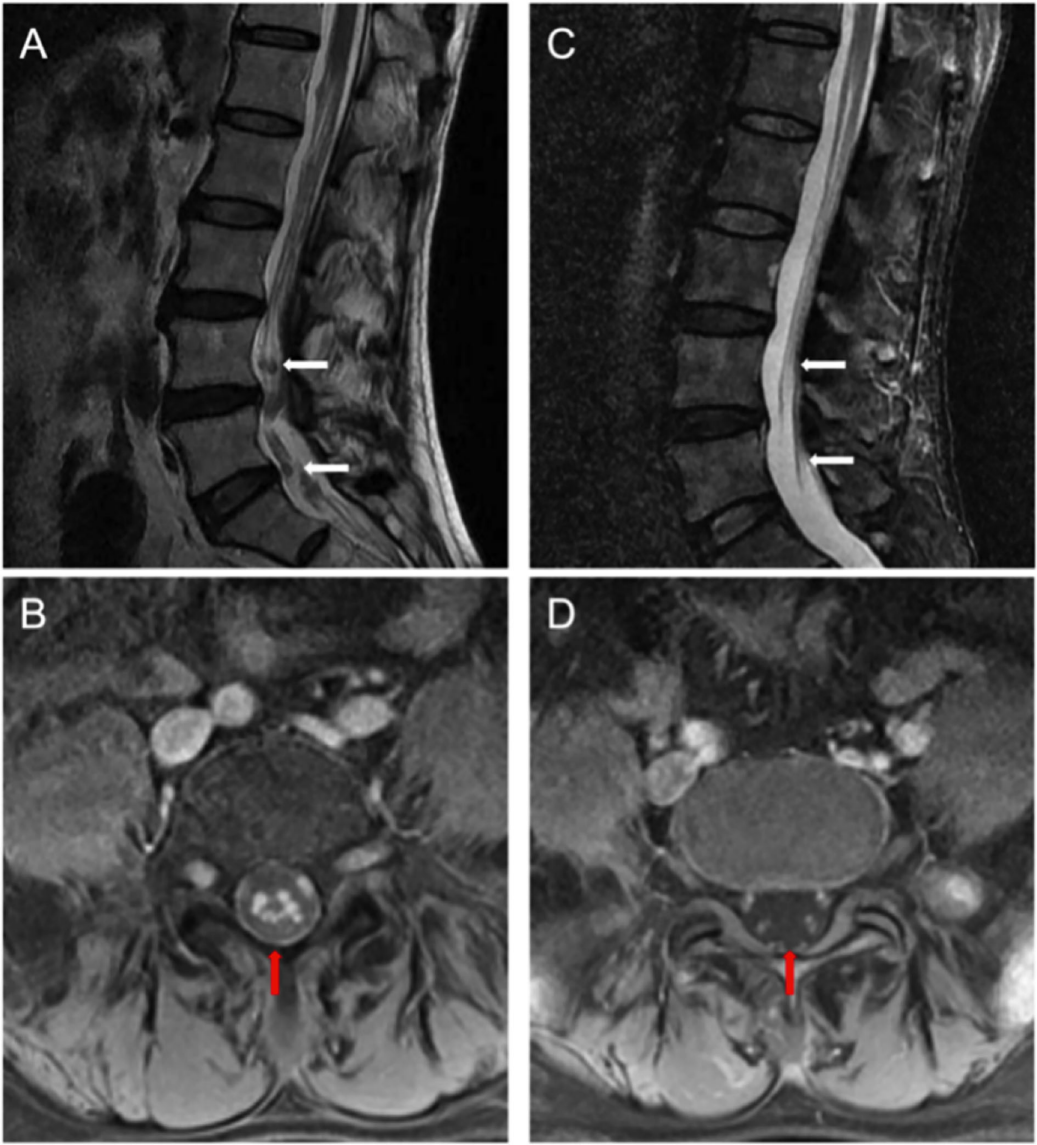
**(A)** Pretreatment lumbar MRI showed multiple diffuse abnormal signal nods in the cauda equina at lumbar and caudal level. **(B)** Enhanced transversal scanning. **(C)** Lesion disappeared in the post-treatment imaging, indicating a complete remission (CR). **(D)** Enhanced transversal scanning also verified complete remission of these lesions after BCMA CAR-T cell therapy [high-resolution T2-weighted image].

Next, morphological analysis showed that the nucleated cells were eliminated in CSF 1 month after CAR-T cell treatment. Imaging evaluation demonstrated that the extramedullary lesion had entirely regressed (**Figures 2F**, **5**) and that he achieved CR with relief of previous manifestations of CES (pain and weakness in both lower limbs, and urinary incontinence).

Unfortunately, plasmacytoma reappeared in his cerebral parenchyma 317 days after infusion, of which he died, at 392 days after infusion, despite of further localized cerebrospinal radiotherapy and anti-CD38 monoclonal antibody treatment.

Most importantly, our report is an unprecedented case of CES by CNS MM, which was successfully treated by BCMA CAR-T therapy. With the installation of the Ommaya reservoir, we could record and track the most detailed CAR-T treatment dynamics data in a CNS-involved patient. The clinical data strongly suggested that BCMA CAR-T cells are capable of entering the blood–brain barrier, amplifying and exerting cytotoxicity in CSF, which is an irreplaceable advantage of BCMA CAR-T cells compared to chemotherapy and surgical treatment. The case demonstrated that BCMA CAR-T cells are effective and safe in multiple myeloma with central nervous system involvement. Furthermore, the latest preclinical trials supported that the intracerebroventricular injection of CAR-T cells may achieve more durable tumor cell eradication for mice with CNS-involved malignancies (26). Hence, we deduced that the intra-cerebroventricular injection of CAR-T cells for patients with CNS involvement *via* an Ommaya reservoir could offer a even more valuable new strategy in the future.

## Data Availability Statement

The original contributions presented in the study are included in the article/supplementary material. Further inquiries can be directed to the corresponding authors.

## Ethics Statement

The studies involving human participants were reviewed and approved by the ethics committee of the First Affiliated Hospital of Zhejiang University. The patients/participants provided their written informed consent to participate in this study. Written informed consent was obtained from the individual(s) for the publication of any potentially identifiable images or data included in this article.

## Author Contributions

YW, YH, and HH designed the study. YW, RH, LW, and HZ analyzed and interpreted the data. YW, YZ, EY, CZ, YH, and HH drafted the article. GW, LY, AJ, YH, and HH provided CAR-T cell treatment and care to patient. All authors contributed to the article and approved the submitted version.

## Funding

This work was supported by Zhejiang Provincial Key Medical Discipline (Medical Tissue Engineering).

## Conflict of Interest

All of the authors declare that the research was conducted in the absence of any commercial or financial relationships that could be construed as a potential conflict of interest.

## Publisher’s Note

All claims expressed in this article are solely those of the authors and do not necessarily represent those of their affiliated organizations, or those of the publisher, the editors and the reviewers. Any product that may be evaluated in this article, or claim that may be made by its manufacturer, is not guaranteed or endorsed by the publisher.
